# Cortical and Subthalamic Nucleus Spectral Changes During Limb Movements in Parkinson's Disease Patients with and Without Dystonia

**DOI:** 10.1002/mds.29057

**Published:** 2022-06-14

**Authors:** Joseph W. Olson, Arie Nakhmani, Zachary T. Irwin, Lloyd J. Edwards, Christopher L. Gonzalez, Melissa H. Wade, Sarah D. Black, Mohammad Z. Awad, Daniel J. Kuhman, Christopher P. Hurt, Bart L. Guthrie, Harrison C. Walker

**Affiliations:** ^1^ Department of Neurology University of Alabama at Birmingham Birmingham Alabama USA; ^2^ Department of Electrical and Computer Engineering University of Alabama at Birmingham Birmingham Alabama USA; ^3^ Department of Neurosurgery University of Alabama at Birmingham Birmingham Alabama USA; ^4^ Department of Biostatistics University of Alabama at Birmingham Birmingham Alabama USA; ^5^ Department of Physical Therapy University of Alabama at Birmingham Birmingham Alabama USA

**Keywords:** Biomarker, Dystonia, Motor Cortex, Parkinson, Spectral Analysis, Subthalamic Nucleus

## Abstract

**Background:**

Dystonia is an understudied motor feature of Parkinson's disease (PD). Although considerable efforts have focused on brain oscillations related to the cardinal symptoms of PD, whether dystonia is associated with specific electrophysiological features is unclear.

**Objective:**

The objective of this study was to investigate subcortical and cortical field potentials at rest and during contralateral hand and foot movements in patients with PD with and without dystonia.

**Methods:**

We examined the prevalence and distribution of dystonia in patients with PD undergoing deep brain stimulation surgery.  During surgery, we recorded intracranial electrophysiology from the motor cortex and directional electrodes in the subthalamic nucleus (STN) both at rest and during self‐paced repetitive contralateral hand and foot movements. Wavelet transforms and mixed models characterized changes in spectral content in patients with and without dystonia.

**Results:**

Dystonia was highly prevalent at enrollment (61%) and occurred most commonly in the foot. Regardless of dystonia status, cortical recordings display beta (13–30 Hz) desynchronization during movements versus rest, while STN signals show increased power in low frequencies (6.0 ± 3.3 and 4.2 ± 2.9 Hz peak frequencies for hand and foot movements, respectively). Patients with PD with dystonia during deep brain stimulation surgery displayed greater M1 beta power at rest and STN low‐frequency power during movements versus those without dystonia.

**Conclusions:**

Spectral power in motor cortex and STN field potentials differs markedly during repetitive limb movements, with cortical beta desynchronization and subcortical low‐frequency synchronization, especially in patients with PD with dystonia. Greater knowledge on field potential dynamics in human motor circuits can inform dystonia pathophysiology in PD and guide novel approaches to therapy. © 2022 The Authors. *Movement Disorders* published by Wiley Periodicals LLC on behalf of International Parkinson and Movement Disorder Society

Dystonia—a twisting, sustained, and often painful involuntary movement—is an understudied motor manifestation of Parkinson's disease (PD).^1^, ^2^, ^3^, ^4^ A hallmark of young‐onset PD, dystonia commonly begins in the foot on the more affected side of the body and spreads to other anatomic locations with disease progression.^5^, ^6^, ^7^, ^8^ Accurate estimates of the prevalence of PD‐related dystonia have been difficult to obtain. Dystonia may be a presenting symptom of PD in younger patients, occurring in 14–50% of patients with young‐onset PD. Patients with PD with the parkin mutation have a higher prevalence rate estimated at around 78%.^9^ About 30% of levodopa‐treated patients with PD have some form of dystonia,^1^ and dystonia severity increases with duration of disease with 2%, 12%, and 56% prevalence rates at <5, 6–9, and >10 years’ duration, respectively.^10^

Deep brain stimulation (DBS) of the subthalamic nucleus (STN) is an effective treatment for dystonia related to PD^11^, ^12^, ^13^; however, little is known about its circuit‐level electrophysiology. More, however, is known about the electrophysiology of primary dystonia. Growing evidence suggests that primary dystonia is marked by excessive theta and alpha oscillations (<13 Hz) in globus pallidus interna at rest, while PD has been associated with excessive power in the beta frequency range (13–30 Hz).^14^, ^15^, ^16^, ^17^, ^18^, ^19^, ^20^, ^21^, ^22^ A recent metaanalysis suggests the STN has similar increases in theta and alpha power at rest in primary dystonia compared with PD, with no significant differences in the beta band.^23^, ^24^, ^25^, ^26^ Resting local field potentials (LFPs) in the primary motor cortex (M1) did not differ significantly for any frequency band in the same metaanalysis.^21^, ^23^, ^27^, ^28^ To our knowledge, no studies have compared cortical and subcortical LFPs in patients with PD with and without dystonia.

Dystonia is commonly worsened by voluntary movements and can emerge spontaneously in other body parts during voluntary movements. Also, primary dystonia presents in many forms (focal, cervical, generalized, etc.), while PD‐related dystonia is most common in the foot.^29^, ^30^ Brain electrophysiology during upper and lower limb movements could help characterize motor circuit contributions to PD symptoms such as dystonia. One recent study reported that lower and upper limb movement onset coincides with high and low beta desynchronization, respectively, in both STN and motor cortex.^31^ Other studies analyzing spontaneous LFPs during resting, sitting, standing, and walking have found mixed results regarding movement‐related STN beta desynchronization.^32^, ^33^, ^34^, ^35^, ^36^ Greater mechanistic knowledge about changes in STN field potentials during specific limb movements could lead to a better understanding of the pathophysiology and novel strategies for therapy.

In this study, we investigate LFPs recorded in parallel from sensorimotor cortex and STN during DBS surgery in patients with PD with and without dystonia. We recorded from an electrocorticography (ECoG) strip over the “hand knob” of sensorimotor cortex and a directional DBS lead placed at dorsolateral STN. Spectral power at rest and during contralateral hand and foot movements in patients with PD, with and without dystonia, was analyzed to test two primary hypotheses: (1) cortical and/or subcortical LFP spectral power differs between hand and foot movements in patients with PD (regardless of dystonia status); and (2) cortical and/or subcortical spectral power differs in patients with PD with versus without dystonia.

## Subjects and Methods

### Recruitment and Enrollment

Participants were recruited and studied prospectively as part of the SUNDIAL (SUbthalamic Nucleus DIrectionAL stimulation) study, a randomized, double‐blind crossover study contrasting directional versus circular unilateral STN DBS for PD (FDA Investigational Device Exemption G‐170063). Dystonia status was not part of the enrollment criteria. Further details on inclusion/exclusion are outlined at ClinicalTrials.gov (https://clinicaltrials.gov/ct2/show/NCT03353688). All subjects provided written informed consent before participation with approval from the institutional review board. We included data from all consecutively enrolled participants.

### Dystonia Rating

We measured PD motor symptoms with normed, validated clinical instruments, including Movement Disorder Society Unified Parkinson's Disease Rating Scale (MDS‐UPDRS) Part III and Part IV item 6 (item 4.6; painful *off* dystonia question). There are no comprehensive dystonia rating scales related specifically to PD; therefore, the Burke‐Fahn‐Marsden (BFM) dystonia scale was used to characterize its severity and anatomic distribution.^37^ These measures were obtained in a presurgery baseline screening visit and longitudinally at 2 and 4 months postsurgery in the practically defined *off* medication state (off dopaminergic medications >12 hours). In addition, we determined whether dystonia was present or absent intraoperatively based on a neurological examination.

### Electrophysiological Recordings

**FIG 1:**
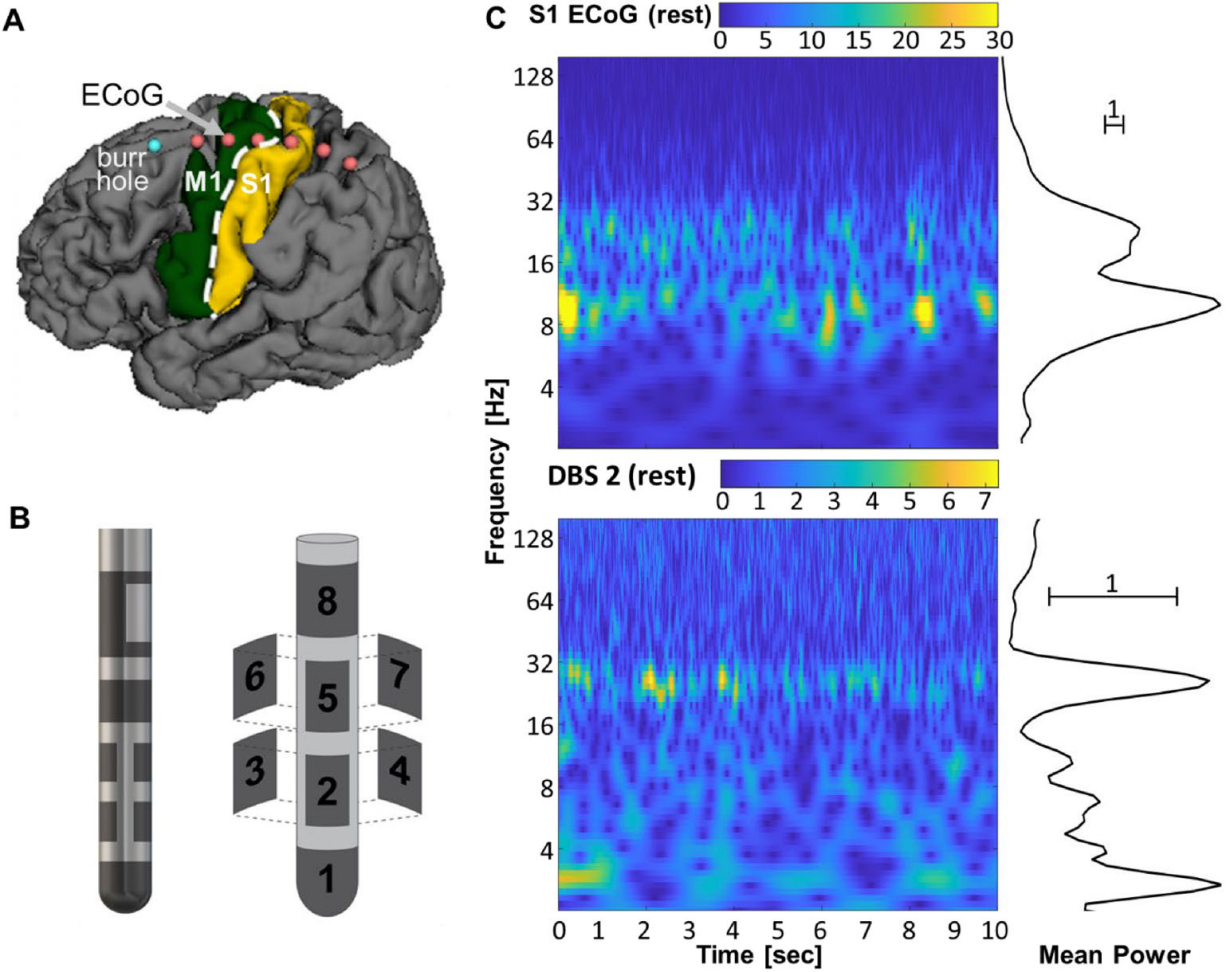
Cortical and subcortical signal recording. (**A**) Primary motor cortex (M1) and primary somatosensory cortex (S1) regions are overlaid on segmented brain from a preoperative magnetic resonance imaging scan. The (ECoG) strip with six contacts is placed over the hand knob area of the primary sensorimotor cortex (contacts are outlined in red). It is inserted through the burr hole (blue) during a surgical deep brain stimulation (DBS) procedure. (**B**) Directional DBS electrode organization with two rings and two rows of three contacts each (1‐3‐3‐1). It is placed along the dorsolateral border of subthalamic nucleus (STN) as part of routine care. (**C**) Resting state wavelet scalogram example from S1 (top) and DBS contact 2 (bottom) for a single participant. The mean spectral power estimated on the right is computed by averaging each row of the scalogram over time.

In our practice, DBS surgeries are conducted unilaterally with the patient awake and *off* dopaminergic medications. We recorded LFPs simultaneously from the DBS lead in its final target location in STN and from a linear six‐contact ECoG strip placed temporarily over the hand knob area of the ipsilateral sensorimotor cortex (Fig. 1A).^38^ Strip placement was well tolerated and did not incorporate diuretics or sedative medications. The directional DBS lead (Boston Scientific's Vercise Cartesia lead) consists of eight contacts arranged into four rows, each separated by 0.5 mm in a 1‐3‐3‐1 configuration (Fig. 1B). The lead is placed such that the dorsolateral border of STN lies between the two middle rows, with contacts 2 and 5 facing anteriorly. We acquired surface electromyography (EMG) recordings from contralateral first dorsal interosseous, flexor carpi radialis, and gastrocnemius muscles. All signals were sampled continuously at 25 kHz using a BrainVision actiCHamp amplifier without filters.

### Behavioral Testing Intraoperative

During intraoperative recording, patients were instructed to rest comfortably over two separate time intervals of 1 minute each. Verbal instructions for moving the contralateral hand or foot were “open and close your hand as big and fast as possible when you are told ‘go’ until the examiner says ‘stop,’” and “repetitively move your toes and ankle when you are told ‘go’ until the examiner says ‘stop.’” Button press signals corresponded to verbal commands from the examiner (“ready,” “set,” “go,” and “stop”). Hand and foot movements were both recorded in nonconsecutive 10‐second blocks, with two repetitions each. Therefore, the total recording time for rest, foot movement, and hand movement conditions was approximately 2 minutes and 40 seconds (with additional transition time for verbal instructions). Rest and movement intervals were validated post hoc based on visualization of EMG recordings.

### Neuroimaging

Preoperative MAGNETOM Prisma magnetic resonance imaging scans were segmented with FreeSurfer (Harvard, Martinos Center for Biomedical Imaging) and registered with intraoperative O‐arm computed tomography for the anatomical localization of ECoG contacts.^39^ We used standard methods in FreeSurfer to parcellate anatomic regions on the cortical surface. These regions included dorsal premotor cortex, supplementary motor area, M1, primary somatosensory cortex (S1), or secondary somatosensory cortex (S2/S3). High‐resolution postoperative computed tomography was obtained to verify anterior orientation of contacts 2 and 5 on the DBS lead using automated software (Brainlab, Munich, Germany). Directional DBS contacts were classified by row and into medial, anterior, and lateral orientations based on implant hemisphere.

### Digital Signal Processing and Analysis

Signal processing was performed with in‐house MATLAB (R2020a; MathWorks, Natick, MA) code and EEGLAB toolbox (University of California San Diego, Swartz Center for Computational Neuroscience).^40^ To verify data integrity, we confirmed normal tissue impedance, visualized the raw continuous signals, and generated spectral plots with continuous Morse wavelet transforms (MATLAB function ‘cwt’) on all channels. Invalid signals (with out‐of‐range impedances) were excluded from subsequent analyses. We separately re‐referenced ECoG and DBS signals, using common average reference within each recording modality. All available signals across recording modalities were analyzed, totaling as many as 14 recording sites plus three EMG electrodes per participant.

We visualized the LFPs in two ways. First, we generated wavelet scalograms for each contact and then averaged the scalograms over time to obtain spectral magnitude (henceforth called power) across relevant frequencies (Fig. 1C). To avoid edge artifacts in the wavelet transform computation, we included 2 seconds before and after the movement block, computed the wavelet transform with the expanded signal, and then removed the 4 seconds for padding. Second, we compared the spectra from each DBS and ECoG location at rest and during hand and foot movements with and without dystonia. Frequency ranges for the field potentials are defined as follows: delta (0.5–4 Hz), theta (4–8 Hz), alpha (8–13 Hz), beta (13–30 Hz), gamma (30–70 Hz), and high‐frequency broadband (hfb) (70–160 Hz). The EMG signal was processed with a 20‐ to 1000‐Hz band‐pass filter and 60‐Hz notch filter, followed by rectification and smoothing with a 250‐ms Gaussian window. Movement pace was estimated from the EMG by finding peaks (MATLAB function ‘findpeaks’) and using the reciprocal of the average time between peaks.

### Statistical Approach

Descriptive statistics, including mean, standard deviation, minimum, maximum, and 95% confidence interval (CI), were computed for LFP spectral power averaged across all valid contacts within each frequency band for foot, hand, and rest. For each frequency band, we used paired *t* tests to compare spectral power during hand and foot movements both with rest and with each other (see Fig. 3A).

To assess spectral differences between patients with or without dystonia (see Fig. 5), we used linear mixed models^41^, ^42^ with dichotomous predictor for dystonia status, fourth‐degree natural polynomials for log‐transformed frequency, and the interaction between dystonia status and polynomials as fixed effects to model the wavelet spectrum averages for all recorded channels. Subject‐specific intercepts were included as random effects. We identified statistically significant differences between spectrum intervals by identifying overlap within 95% CIs. Linear mixed models were fitted for foot, hand, and rest, and CIs were compared based on UPDRS item 4.6, BFM, and surgical examination. No statistical corrections for multiple comparisons were performed.

### Data Sharing

All data are available on Data Archive for the Brain Initiative (https://dabi.loni.usc.edu/home).

## Results

### Demographics

We studied intraoperative LFPs in 28 of 38 (74%) participants who met entry criteria for the SUNDIAL trial. Exclusions related to the following: ≤30% improvement UPDRS Part III *on* versus *off* PD medications (*n* = 4), Beck Depression Inventory >10 (*n* = 1), multidisciplinary committee recommended globus pallidus rather than STN target (*n* = 2), medical condition requiring future magnetic resonance imaging (*n* = 1), health insurance refused payment for surgery with an investigational device (*n* = 1), and voluntary withdrawal (*n* = 1). Of the 28 participants with recorded LFPs, 3 had corrupted or missing data, yielding 25 participants for subsequent analyses.

### Spectral Perturbations from Rest During Hand and Foot Movements

**FIG 2:**
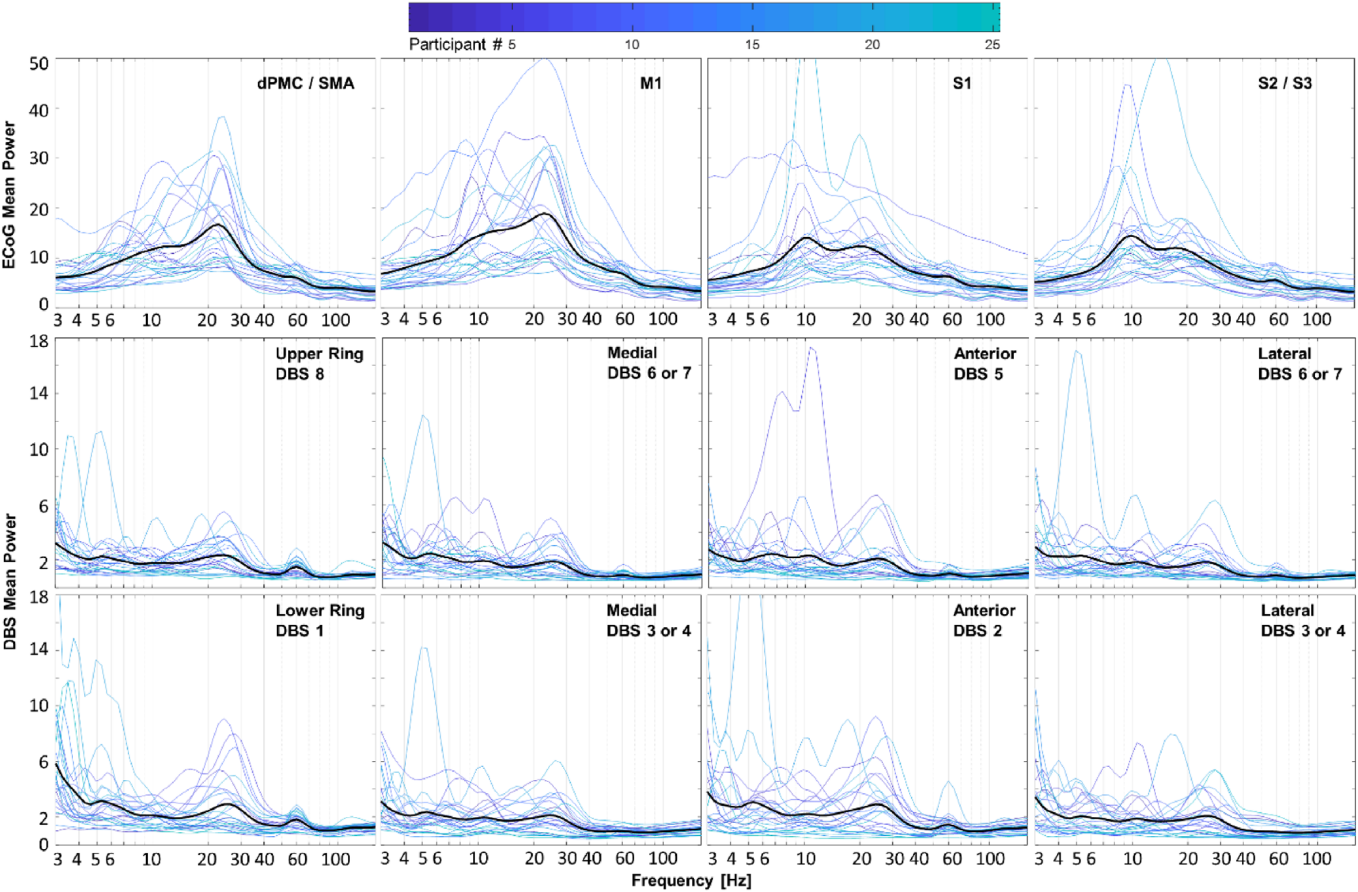
Cortical and subcortical field potentials at rest in participants with Parkinson's disease. Spectral power from all electrode contacts over the ipsilateral cortex (first row) and the subthalamic nucleus (STN) region (second and third rows) are categorized by anatomical location obtained from imaging, regardless of dystonia status. For each location, there is one plot per participant color‐coded by participant number. Means are represented by darker black lines. Beta frequency power is present and relatively large in essentially all electrocorticography (ECoG) contacts, whereas lower beta power is present in most, but not all, deep brain stimulation (DBS) contacts, along with greater relative power at lower frequencies. dPMC, dorsal premotor cortex; M1, primary motor cortex; S1, primary somatosensory cortex; SMA, supplementary motor area.

**FIG 3:**
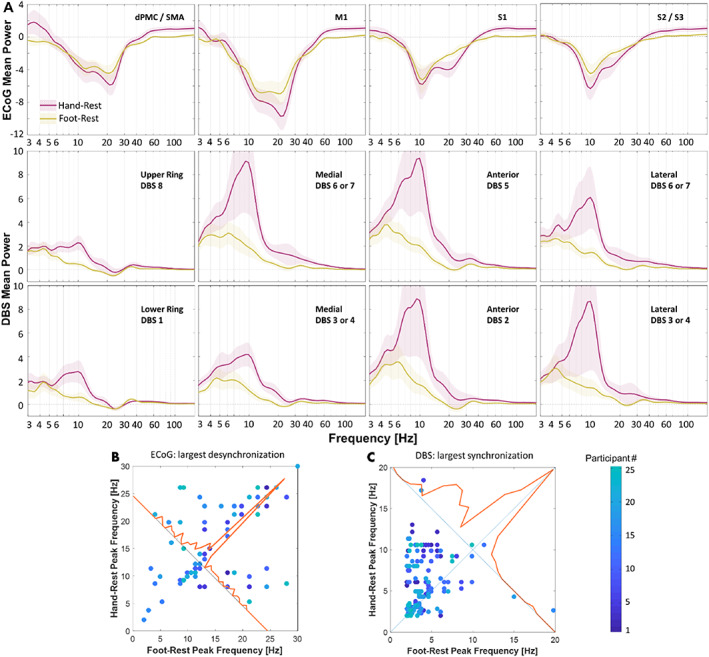
Cortical and subcortical spectral perturbations in participants with Parkinson's disease (PD) during continuous, repetitive hand and foot movements minus rest. (**A**) Spectral power for all participants (*n* = 25) and recording sites are summarized in row 1 for sensorimotor cortex and rows 2 and 3 for dorsal and ventral deep brain stimulation (DBS) contacts (mean across participants ± standard error). Red and yellow traces correspond to hand and foot signals, respectively. (**B, C**) Frequencies that displayed the strongest deviations during movement versus rest in cortex and subthalamic nucleus (STN; ie, either desynchronization or synchronization). If the spectral perturbations are identical, observations will appear on or near the unity line (diagonal line). (**B**) Frequencies corresponding to power minima in dorsal premotor cortex/supplementary motor area, primary motor cortex (M1), primary somatosensory cortex (S1), and S2/S3 contacts during foot versus hand movements. (**C**) Frequencies corresponding to power maxima from all DBS contacts during foot versus hand movements. ECoG, electrocorticography.

The EMG recordings of all 25 participants were obtained to verify their resting intervals. Spectral analyses of resting field potentials demonstrated peaks in the beta frequency range (13–30 Hz) in both sensorimotor cortex and STN, with approximately five times the magnitude at cortical recording sites (Fig. 2). To isolate changes in spectral power related to movement, we subtracted resting spectra from movement spectra within each channel (Fig. 3A). Cortical LFPs demonstrate broadband power changes during repetitive voluntary hand and foot movements compared with rest. Alpha and beta power of cortical LFPs decrease markedly during both movements, whereas hfb power increases during hand movements only (foot—theta: −1.0 ± 1.9, *P* = 0.014; alpha: −4.0 ± 3.8, *P* < 0.001; beta: −3.4 ± 2.3, *P* < 0.001; gamma: −0.6 ± 0.9, *P* = 0.004; hand—alpha: −4.8 ± 5.0, *P* < 0.001; beta: −4.6 ± 3.6, *P* < 0.001; hfb: 1.0 ± 1.7, *P* = 0.006; mean ± standard deviation, *t* test). Spectral power differs significantly between foot and hand movements in the beta, gamma, and high‐frequency bands (beta: 1.2 ± 2.2, *P* = 0.014; gamma: −0.6 ± 1.2, *P* = 0.028; hfb: −0.9 ± 1.1, *P* < 0.001). Interestingly, spectral power in STN signals during repetitive limb movements displayed an almost opposite behavior. Theta, alpha, and low beta spectral power increased during movements compared with rest, while the higher beta power increased slightly or did not change (foot—delta: 2.0 ± 1.7, *P* < 0.001; theta: 2.3 ± 2.9, *P* < 0.001; alpha: 1.3 ± 2.3, *P* = 0.010; gamma: 0.2 ± 0.2, *P* < 0.001; hfb: 0.1 ± 0.0, *P* < 0.001; hand—delta: 2.6 ± 2.3, *P* < 0.001; theta: 4.1 ± 5.8, *P* = 0.002; alpha: 5.5 ± 11.8, *P* = 0.029; gamma: 0.4 ± 0.8, *P* = 0.012; hfb: 0.1 ± 0.2, *P* < 0.001). Furthermore, spectral power during foot and hand movements differed from each other in theta, alpha, and hfb frequency ranges (theta: −1.7 ± 3.6, *P* = 0.023; alpha: −4.2 ± 9.8, *P* = 0.043; hfb: −0.1 ± 0.2, *P* = 0.023).

In addition to measuring spectral power, we also characterized which peak frequencies displayed the most prominent changes during limb movements. We measured frequencies with the maximal desynchronization during hand and foot movements versus rest in the ECoG signals (Fig. 3B). Given that the STN field potentials were inverted and showed increases in spectral power, we instead measured frequencies with maximal synchronizations (Fig. 3C). In most cases, maximal cortical desynchronization occurred at similar frequencies during foot versus hand movements (14.4 ± 6.6 and 15.8 ± 6.7 Hz, respectively; mean ± standard deviation; *P* = 0.460, paired *t* test). In contrast, maximal synchronization in the STN region occurred at lower peak frequencies during foot versus hand movements (4.2 ± 2.9 and 6.0 ± 3.3 Hz, respectively; *P* = 0.046).

We found no statistically significant correlation between LFP frequency and movement rate during hand or foot movements (Supporting Information Fig. S1).

### Dystonia Prevalence and Clinical Features

**FIG 4:**
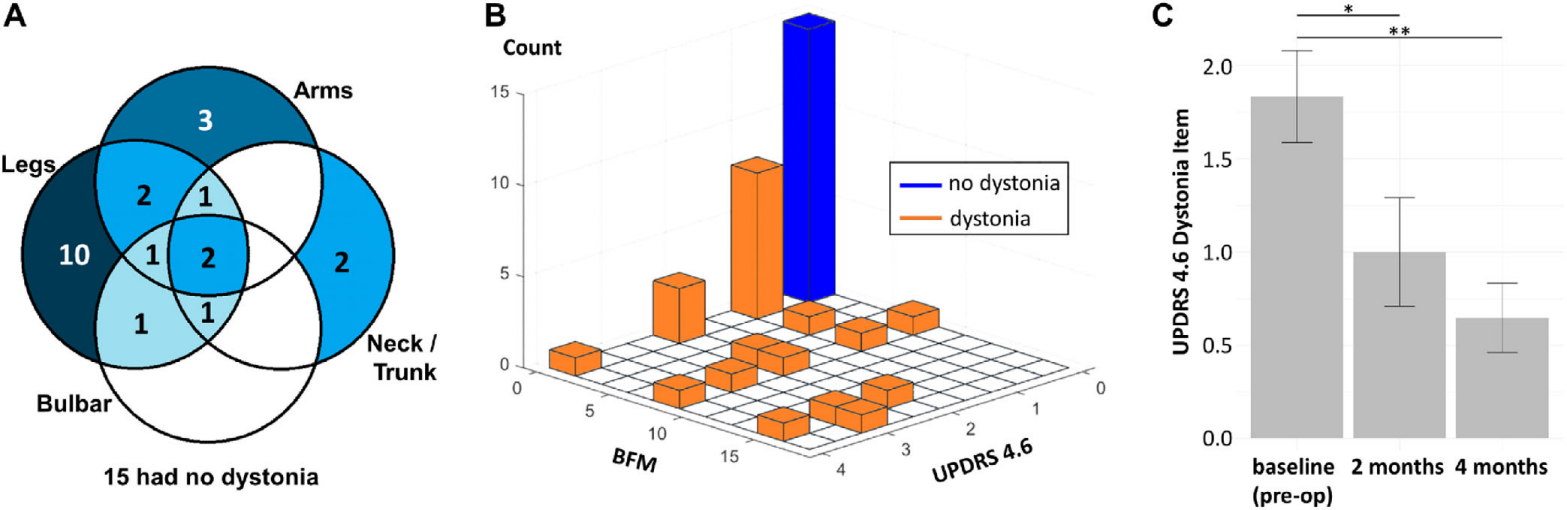
Prevalence and anatomic distribution of dystonia in participants with Parkinson's disease (PD) evaluated for deep brain stimulation (DBS) surgery during baseline visit and clinical outcomes. (**A**) Venn diagram of anatomic distribution of dystonia clustered into four groups (arms, legs, bulbar, neck/trunk). The number in each circle intersection denotes the number of participants who had the corresponding parts affected by the dystonia (self‐reported or discovered by Burke‐Fahn‐Marsden [BFM] dystonia rating scale). Fifteen of 38 participants had no dystonia at baseline. (**B**) Three‐dimensional histogram of Unified Parkinson's Disease Rating Scale (UPDRS) dystonia item 4.6 (painful dystonia scale) versus BFM total score. Orange bars denote participants with dystonia, and the blue bar denotes participants with no dystonia. (**C**) Dystonia motor outcomes after DBS. Error bars indicate standard error. Both 2 and 4 months are significant from baseline (*P* = 0.002; *P* < 0.001).

Dystonia status was assessed based on participant report (UPDRS item 4.6), the BFM score at a motor screening visit, and neurological examination during DBS surgery. We provide all available behavioral data on PD‐related dystonia, even in participants who were screen failures for the intraoperative component of the study, to provide a more comprehensive accounting of dystonia phenomenology in patients with PD. In total, dystonia was present based on history, examination, or both in 23 of 38 participants (61%). Dystonia occurred most often, but not exclusively, in the foot (18/23, 78%) (Fig. 4A). Among survey responses from baseline screening, 8 of 17 participants (47%) report worsening of dystonia during various movements, whereas other reported dystonia primarily at rest (Supporting Information Table S1). During surgery, 8 of 25 participants (32%) had subjective or visible dystonia *off* medications. Although UPDRS item 4.6 correlates linearly with BFM total score (F = 8.337, *P* = 0.006, adjusted *R* ^2^ = 0.114) (Fig. 4B), of the 22 participants with dystonia based on UPDRS 4.6, only 12 (45%) had dystonia *off* medications at their screening visit based on BFM, illustrating the transient nature of dystonia in PD. Interim analyses provide evidence that unilateral STN DBS improves dystonia symptoms, based on changes in UPDRS item 4.6 at 2 and 4 months postoperatively versus preoperative baseline (*F* = 24.127, *P* < 0.001, linear mixed model with dependent variable UPDRS item 4.6 and fixed effect time, subject‐specific random intercept) (Fig. 4C). UPDRS Part III total score *off* medications shows clinically significant motor improvement of 33.7%, 32.6%, and 37.5% at 2, 4, and 6 months versus preoperative baseline (baseline score 49.2 ± 2.38 reduced by 16.6 ± 1.6, 16.0 ± 1.6, and 18.6 ± 1.6, *P* < 0.001, respectively, linear mixed effects model).^43^

### Electrophysiological Biomarkers of Dystonia in PD

**FIG 5:**
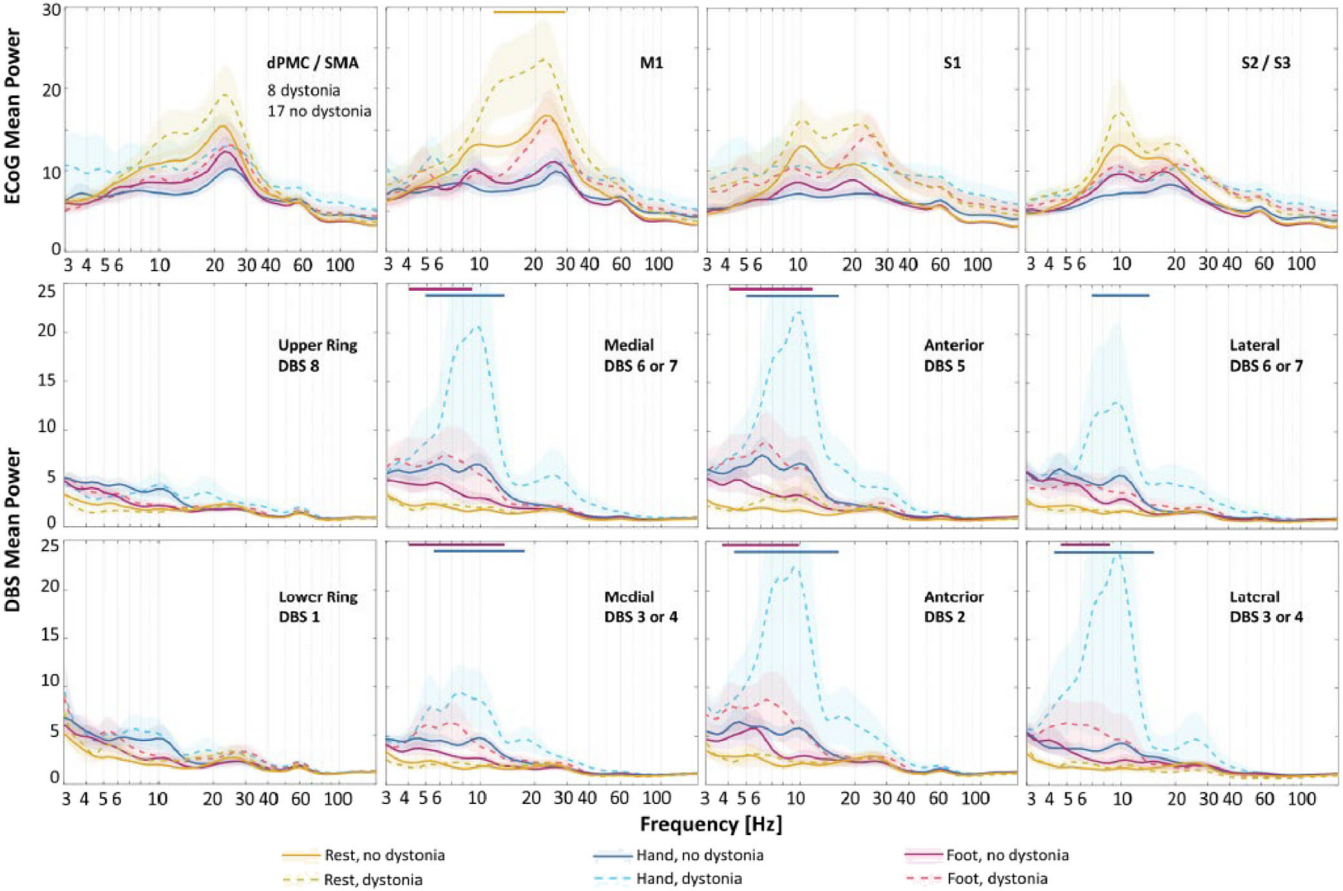
Cortical and subcortical spectral perturbations during rest and hand and foot movements in participants with Parkinson's disease (PD) with versus without dystonia during deep brain stimulation (DBS) surgery. Spectral power from electrocorticography (ECoG) contacts in ipsilateral cortical (first row) and DBS contacts in subthalamic nucleus (STN) region (second row) are categorized by the presence or absence of dystonia during DBS surgery (mean ± standard error). Dashed and solid lines represent average spectral power in patients with PD with and without dystonia, respectively. Yellow, blue, and red colors correspond to rest, hand, and foot movements. Shaded standard errors are for visual purposes only and do not necessarily reflect the statistical confidence intervals of nonnormal variance. Horizontal colored bars above the plots correspond to frequency intervals with statistically significant differences. dPMC, dorsal premotor cortex; M1, primary motor cortex; S1, primary somatosensory cortex; SMA, supplementary motor area.

All patients were classified as having or not having dystonia based on neurological examination during surgery. Wavelet spectrum averages for each channel were plotted at rest and during hand and foot movements (Fig. 5). There was a greater beta power (13–29 Hz) in M1 at rest in patients with PD with versus without dystonia (95% CI). There were no other significant differences in spectral power between patients with PD with and without dystonia at other cortical regions or frequency range across conditions at our level of statistical power. We observed no significant differences in STN power at rest in patients with PD with versus without dystonia. During both foot and hand movements, we detected greater STN low‐frequency power (~4–12 and ~5–15 Hz, respectively) in patients with PD with dystonia in directional DBS contacts only (95% CIs). We also compared spectral power of patients with PD with versus without dystonia using UPDRS item 4.6 and BFM rating scales (Supporting Information Fig. S2). Group differences were greatest based on the presence or absence of dystonia at the time of the recordings during surgery.

## Discussion

In this article, we report three primary findings, each related primarily to activity in the theta and alpha frequency ranges. First, we use intraoperative LFPs to provide evidence that patients with PD with versus without dystonia display greater low‐frequency STN power during limb movements and greater beta power in M1 at rest (Fig. 5). Second, regardless of dystonia status, foot movements increased STN spectral power predominantly in the theta range, whereas contralateral hand movements yielded greater synchrony at alpha frequencies (Fig. 3). Finally, sensorimotor cortex and basal ganglia field potentials differ markedly during repetitive, naturalistic voluntary movements, with sustained beta desynchronization in cortex and increased low‐frequency power in the STN (Fig. 3).

### Dystonia Pathophysiology in Subcortical and Cortical Field Potentials

Although we are unaware of prior studies on this topic in patients with PD, studies on primary dystonia suggest excessive theta and alpha power in basal ganglia at rest compared with PD (regardless of their dystonia status). We did not identify similar differences in our sample at rest but did observe excessive STN theta and alpha power during limb movements (Fig. 5). Dystonia symptoms often worsen with voluntary movements, such that dystonia pathophysiology might be more pronounced or easily detected in STN during movements (Supporting Information Table S1). We also found increased M1 beta power at rest in patients with PD with versus without dystonia, where other groups have used TMS to identify abnormal short‐term plasticity in patients with both primary dystonia and PD.^44^ Notably, we found increases in hfb power during hand movements only, which confirms placement of the ECoG strip over the hand area of the motor cortex^45^, ^46^ (Fig. 3).

### Low‐Frequency STN Activity During Upper and Lower Limb Movements

In the STN, repetitive limb movements yielded broadband increases in low‐frequency power, with foot and hand movements manifesting primarily in the theta and alpha frequency ranges, respectively (Fig. 3). Although this differs somewhat from a study analyzing LFPs time‐locked to movement onset,^31^ others have implicated low‐frequency activity in STN during lower limb movements as well.^32^, ^34^ Although some argue that these low‐frequency signals in basal ganglia represent movement artifacts,^34^ we and others^32^ contend that they likely arise from brain signals for several reasons. First, ECoG and DBS signals in our paradigm show opposite changes in spectral power during movements versus rest (Figs. 3 and 5), yet the wires that transmit the signals are bundled together and are sampled by the same amplifier. Second, we observed no correlations between peak frequencies in the LFPs and the pace or rate of movements across participants (Supporting Information Fig. S1).

### Beta Desynchronization Is Greater in the Sensorimotor Cortex than in STN

Consistent with prior studies, we found prominent beta band frequencies at rest in the cortex, along with large and sustained beta desynchronizations during voluntary movements.^47^ Resting beta power was also observed in STN, although it was considerably smaller in power and present less consistently (Fig. 2). In agreement with a similar recent study, we did not observe beta desynchronization during repetitive movements (Fig. 3).^48^ These results may differ from prior studies reporting beta desynchronization in STN at movement onset because our paradigm studied naturalistic, self‐paced, repetitive movements rather than cued single movements. Repetitive or complex movements can display beta‐rebound phenomena,^49^, ^50^ and “microlesion” effects from acute lead placement might alter the local responses as well.^51^ Regardless, beta desynchronization in cortex was more robust and sustained during sustained repetitive movements versus in STN.

### Clinical Translational Significance

Dystonia is an underrecognized symptom of PD, with historical prevalence estimates at approximately 30%.^1^ Although we observed dystonia during surgery in 31% of our sample, other available measures indicate a total prevalence of at least 61%. Another group reported higher dystonia prevalence (71%) in patients with PD undergoing DBS surgery as well.^11^ Underestimates could arise from the fluctuating nature of dystonia, lack of data capture in routine assessments, and/or lack of recognition of dystonia symptoms.

Another contributor is likely sampling bias, such that patients with PD with dystonia are more likely to receive DBS than those without. Given its painful nature and relatively high prevalence in this group of patients, additional electrophysiology studies on PD‐related dystonia are warranted. Simultaneous cortical and directional subcortical field potential recordings in this study provide new pathophysiological knowledge on spectral perturbations during repetitive limb movements in patients with PD with and without dystonia. In addition, multimodal recordings from this study could inform development of emerging adaptive DBS technologies, given that M1 and basal ganglia are leading candidate regions for closed‐loop sensing.^52^

### Study Limitations

Our study has some potential limitations. Time and physical constraints during surgery sometimes limit data collection, and acute implant of the DBS lead introduces microlesion effects that could alter the electrophysiology.^51^ Therefore, elements of these studies should be repeated with implanted devices with sensing capabilities in the chronic setting. Dystonia was most commonly present in the foot, yet our ECoG placements were over the hand knob of the motor cortex. Although we did see increased beta power at rest in patients with dystonia in M1, this nevertheless might have limited our view of cortical physiology related to dystonia. We used EMG recordings to characterize movement states, but future studies would likely benefit from video recordings to better capture time‐locked movement dynamics. Although our sample is considerably larger than most prior efforts in the field, this is an initial implementation of our analytic methods with these broadband signals. As such, no corrections for multiple comparisons were used. Thus, our results should be interpreted with some caution.

## Conclusions

Spectral perturbations in M1 and STN field potentials differ substantially during repetitive arm and leg movements, with prominent beta desynchronization in cortex and increased theta and alpha power in STN, especially in patients with PD with dystonia. Greater knowledge on field potential dynamics in human motor circuits promise to inform dystonia pathophysiology in PD and guide novel approaches to therapy.

## Author Roles

A. Experimental design.

B. Data collection.

C. Data analysis and/or creating figures.

D. Writing of manuscript.

E. Edits of manuscript.

Joseph W. Olson: B, C, D, E

Arie Nakhmani: A, C, D, E

Zachary T. Irwin: A, B, E

Lloyd J. Edwards: C, D, E

Christopher L. Gonzalez: B, C, E

Melissa H. Wade: B, E

Sarah D. Black: C, E

Mohammad Z. Awad: B, E

Daniel J. Kuhman: A, B, E

Christopher P. Hurt: A, B, E

Bart L. Guthrie: B, E

Harrison C. Walker: A, B, C, D, E

## Financial Disclosures (Past 12 months)

Stock Ownership in medically related fields: No authors.

Intellectual Property Rights: No authors.

Consultancies: No authors.

Expert Testimony: No authors.

Advisory Boards: No authors.

Employment: UAB (all authors).

Partnerships: No authors.

Inventions: No authors.

Contracts: No authors.

Honoraria: No authors.

Royalties: No authors.

Patents: No authors

Grants: National Institutes of Health, The Michael J. Fox Foundation.

Other: No authors.

## Supporting information


**Figure S1.** Correlation between the movement pace and spectral extrema. Above are frequencies of maximum cortical desynchronization during movement compared to rest (Fig. 3). For each cortical region, Pearson correlation is given with p‐values not adjusted for multiple comparisons. Below are frequencies of maximum subcortical synchronization during movement compared to rest.

**Figure S2.** Averaged cortical and subcortical spectral perturbations during rest and limb movements in PD participants with versus without dystonia determined by different dystonia rating scales. Spectral power from all electrode contacts over ipsilateral sensorimotor cortex (first row) and STN region (second row) are categorized by presence/absence of dystonia historically (UPDRS 4.6), at pre‐operative baseline assessment (BFM), and during DBS surgery (mean ± standard error). Dashed and solid lines represent average spectral power in PD patients with and without dystonia, respectively. Yellow, blue, and red colors correspond to rest, hand, and foot movements. Shaded standard errors are for visual purposes only and do not necessarily reflect the statistical confidence intervals of non‐normal variance. Horizontal colored bars above the plots correspond to frequency intervals with statistically significant differences. During foot movement, we observed statistically significant differences (p < 0.05) between STN low frequency power in PD patients with vs without dystonia when using BFM (4–12 Hz) and surgical exam (4–11 Hz) but not UPDRS. Significant differences (p < 0.05) in STN power were also observed during hand movement when using surgical exam classification (5–15 Hz). No significant differences in STN power were observed during rest for any dystonia classification. Furthermore, there was a significant difference (p < 0.05) in cortical spectral power during hand movement using surgical exam classification (3–25 Hz). Otherwise, there were no observed differences in cortical spectral power between PD patients with and without dystonia.

**Table S1.** Patient experiences with dystonia surveyed pre‐surgery.

## Data Availability

All data is available on the Data Archive for the Brain Initiative (DABI) at https://dabi.loni.usc.edu/home.

## References

[1] Kidron D , Melamed E . Forms of dystonia in patients with Parkinson's disease. Neurology 1987;37(6):1009–1011. 3587617 10.1212/wnl.37.6.1009

[2] Tolosa E , Compta Y . Dystonia in Parkinson's disease. J Neurol 2006;253:VII/7–VII/13. 10.1007/s00415-006-7003-6 17131231

[3] Tolosa E , Marti MJ , Compta Y . Dystonic symptoms associated with parkinsonian disorders. Handbook of Dystonia 2012;276–296.

[4] Shetty AS , Bhatia KP , Lang AE . Dystonia and Parkinson's disease: what is the relationship? Neurobiol Dis 2019;132:104462. 10.1016/j.nbd.2019.05.001 31078682

[5] Lees AJ , Hardie RJ , Stern GM . Kinesigenic foot dystonia as a presenting feature of Parkinson's disease. J Neurol Neurosurg Psychiatry 1984;47(8):885 10.1136/jnnp.47.8.885 PMC1027959 6470733

[6] LeWitt PA , Burns RS , Newman RP . Dystonia in untreated parkinsonism. Clin Neuropharmacol 1986;9(3):293–297. 3719574 10.1097/00002826-198606000-00007

[7] Jankovic J , Tintner R . Dystonia and parkinsonism. Parkinsonism Relat Disord 2001;8(2):109–121. 11489676 10.1016/s1353-8020(01)00025-6

[8] Gershanik OS . Early onset parkinsonism. Frontiers in Bioscience‐Landmark 2003;8(6):568–578. 10.2741/1100 12700083

[9] Gershanik OS , Leist A . Juvenile onset Parkinson's disease. Advances in Neurology 1987;45:213–216. 3825691

[10] Schrag A , Quinn N . Dyskinesias and motor fluctuations in Parkinson's disease: a community‐based study. Brain 2000;123(11):2297–2305. 11050029 10.1093/brain/123.11.2297

[11] Krack P , Batir A , Van Blercom N , et al. Five‐year follow‐up of bilateral stimulation of the subthalamic nucleus in advanced Parkinson's disease. N Engl J Med 2003;349(20):1925–1934. 10.1056/NEJMoa035275 14614167

[12] Krack P , Pollak P , Limousin P , Benazzouz A , Deuschl G , Benabid A‐L . From off‐ period dystonia to peak‐dose chorea: the clinical spectrum of varying subthalamic nucleus activity. Brain 1999;122:1133–1146. 10356065 10.1093/brain/122.6.1133

[13] Detante O , Vercueil L , Krack P , Chabardes S , Benabid A‐L , Pollak P . Off‐period dystonia in Parkinson's disease but not generalized dystonia is improved by high‐ frequency stimulation of the subthalamic nucleus. Adv Neurol 2004;94:309–314. 14509688

[14] Neumann W‐J , Turner RS , Blankertz B , Mitchell T , Kuhn AA , Richardson RM . Toward electrophysiology‐based intelligent adaptive deep brain stimulation for movement disorders. Neurotherapeutics 2019;16:105–118. 30607748 10.1007/s13311-018-00705-0 PMC6361070

[15] Silberstein P , Kuhn AA , Kupsch A , et al. Patterning of globus pallidus local field potentials differs between Parkinson's disease and dystonia. Brain 2003;126(12):2597–2608. 12937079 10.1093/brain/awg267

[16] Chen CC , Kuhn AA , Trottenberg T , Kupsch A , Schneider G‐H , Brown P . Neuronal activity in globus pallidus interna can be synchronized to local field potential activity over 3–12 Hz in patients with dystonia. Exp Neurol 2006;202:480–486. 16930593 10.1016/j.expneurol.2006.07.011

[17] Liu X , Wang S , Yianni J , et al. The sensory and motor representation of synchronized oscillations in the globus pallidus in patients with primary dystonia. Brain 2008;131:1562–1573. 18487278 10.1093/brain/awn083

[18] Neumann W‐J , Horn A , Ewert S , et al. A localized pallidal physiomarker in cervical dystonia. Ann Neurol 2017;82(6):912–924. 29130551 10.1002/ana.25095

[19] Barow E , Neumann W‐J , Brucke C , et al. Deep brain stimulation suppresses pallidal low frequency activity in patients with phasic dystonic movements. Brain 2014;137(11):3012–3024. 25212852 10.1093/brain/awu258 PMC4813762

[20] Piña‐Fuentes D , van Zijl JC , van Dijk JMC , et al. The characteristics of pallidal low‐frequency and beta bursts could help implementing adaptive brain stimulation in the parkinsonian and dystonic internal globus pallidus. Neurobiol Dis 2019;121:47–57. 30227227 10.1016/j.nbd.2018.09.014

[21] Wang DD , de Hemptinne C , Miocinovic S , Ostrem JL , Galifianakis NB , San Luciano M , Starr PA . Pallidal deep‐brain stimulation disrupts Pallidal Beta oscillations and coherence with primary motor cortex in Parkinson's disease. Neurobiol Dis 2018;38(19):4556–4568. 10.1523/JNEUROSCI.0431-18.2018 PMC5943981 29661966

[22] Lofredi R , Neumann W‐J , Brucke C , Heubl J , Krauss JK , Schneider G‐H , Kuhn AA . Pallidal beta bursts in Parkinson's disease and dystonia. Mov Disord 2019;34(3):420–424. 30440096 10.1002/mds.27524

[23] Piña‐Fuentes D , van Zijl VDJMCDGJC , van Laar T , Tijssen MAJ , Beudel M . Direct comparison of oscillatory activity in the motor system of Parkinson's disease and dystonia: a review of the literature and meta‐analysis. Clin Neurophysiol 2019;130(6):917–924. 30981177 10.1016/j.clinph.2019.02.015

[24] Wang DD , de Hemptinne C , Miocinovic S , et al. Subthalamic local field potentials in Parkinson's disease and isolated dystonia: an evaluation of potential biomarkers. Neurobiol Dis 2016;89:213–222. 26884091 10.1016/j.nbd.2016.02.015 PMC4790450

[25] Geng X , Zhang J , Jiang Y , et al. Comparison of oscillatory activity in subthalamic nucleus in Parkinson's disease and dystonia. Neurobiol Dis 2017;98:100–107. 27940307 10.1016/j.nbd.2016.12.006 PMC5321603

[26] Neumann W‐J , Huebl J , Brucke C , Ruiz MH , Kupsch A , Schneider G‐H , Kuhn AA . Enhanced low‐frequency oscillatory activity of the subthalamic nucleus in a patient with dystonia. Mov Disord 2012;27(8):1063–1066. 22700436 10.1002/mds.25078

[27] Crowell AL , Ryapolova‐Webb ES , Ostrem JL , Galifianakis NB , Shimamoto S , Lim DA , Starr PA . Oscillations in sensorimotor cortex in movement disorders: an electrocorticography study. Brain 2012;135(2):615–630. 22252995 10.1093/brain/awr332 PMC3281473

[28] Miocinovic S , de Hemptinne C , Qasim S , Ostrem JL , Starr PA . Patterns of cortical synchronization in isolated dystonia compared with Parkinson disease. JAMA Neurol 2015;72(11):1244–1251. 26409266 10.1001/jamaneurol.2015.2561 PMC4892933

[29] Phukan J , Albanese A , Gasser T , Warner T . Primary dystonia and dystonia‐plus syndromes: clinical characteristics, diagnosis, and pathogenesis. Lancet Neurol 2011;10(12):1074–1085. 22030388 10.1016/S1474-4422(11)70232-0

[30] Albanese A , Bhatia K , Bressman SB , et al. Phenomenology and classification of dystonia: a consensus update. Mov Disord 2014;28(7):863–873. 10.1002/mds.25475 PMC3729880 23649720

[31] Tinkhauser G , Shah SA , Fischer P , et al. Electrophysiological differences between upper and lower limb movements in the human subthalamic nucleus. Clin Neurophysiol 2019;130(5):727–738. 30903826 10.1016/j.clinph.2019.02.011 PMC6487671

[32] Wang DD , Choi JT . Brain network oscillations during gait in Parkinson's disease. Front Hum Neurosci 2020;14:568703. 10.3389/fnhum.2020.568703 PMC7645204 33192399

[33] Quinn EJ , Blumenfeld Z , Velisar A , et al. Beta oscillations in freely moving Parkinson's subjects are attenuated during deep brain stimulation. Mov Disord 2015;30(13):1750–1758. 26360123 10.1002/mds.26376

[34] Hell F , Plate A , Mehrkens JH , Bötzel K . Subthalamic oscillatory activity and connectivity during gait in Parkinson's disease. Neuroimage Clin 2018;19:396–405. 30035024 10.1016/j.nicl.2018.05.001 PMC6051498

[35] Arnulfo G , Pozzi NG , Palmisano C , et al. Phase matters: a role for the subthalamic network during gait. PLoS One 2018;13(6):e0198691. 10.1371/journal.pone.0198691 PMC5991417 29874298

[36] Neuville RS , Petrucci MN , Wilkins KB , et al. Differential effects of pathological Beta burst dynamics between Parkinson's disease phenotypes across different movements. Front Neurosci 2021;15:733203. 10.3389/fnins.2021.733203 PMC8631908 34858125

[37] Burke RE , Fahn S , Marsden CD , Bressman SB , Moskowitz C , Friendman J . Validity and reliability of a rating scale for the primary torsion dystonias. Neurology 1985;35(1):73–77. 3966004 10.1212/wnl.35.1.73

[38] Nishibayashi H , Ogura M , Kakishita K , Tanaka S , Tachibana Y , Atsushi Nambu KH , Itakura T . Cortically evoked responses of human Pallidal neurons recorded during stereotactic neurosurgery. Mov Disord 2011;26(3):469–476. 21312279 10.1002/mds.23502

[39] Fischl B . FreeSurfer. Neuroimage 2012;62(2):774–781. 22248573 10.1016/j.neuroimage.2012.01.021 PMC3685476

[40] Delorme A , Makeig S . EEGLAB: an open source toolbox for analysis of single‐trial EEG dynamics including independent component analysis. J Neurosci Methods 2004;134:9–21. 15102499 10.1016/j.jneumeth.2003.10.009

[41] Cheng J , Edwards L , Maldonado‐Molina M , Komro K , Muller K . Real longitudinal data analysis for real people: building a good enough mixed model. Stat Med 2010;29:504–520. 20013937 10.1002/sim.3775 PMC2811235

[42] Edwards L . Modern statistical techniques for the analysis of longitudinal data in biomedical research. Pediatr Pulmonol 2000;30(4):330–344. 11015135 10.1002/1099-0496(200010)30:4<330::aid-ppul10>3.0.co;2-d

[43] Duffus S , Chukwueke U , Strowd R , et al. Unilateral vs. bilateral subthalamic stimulation in Parkinson's disease. Neurology 2015;84(14):P1.168.

[44] Udupa K , Chen R . Motor cortical circuits in Parkinson disease and dystonia. Handbook of Clinical Neurology. Vol. 161. 2019:167–186. 31307598 10.1016/B978-0-444-64142-7.00047-3

[45] Leszczyński M , Barczak A , Kajikawa Y , et al. Dissociation of broadband high‐frequency activity and neuronal firing in the neocortex. Sci Adv 2020;6(33):eabb0977. 10.1126/sciadv.abb0977 PMC7423365 32851172

[46] Merrick CM , Dixon TC , Breska A , et al. Left hemisphere dominance for bilateral kinematic encoding in the human brain. eLife 2022;11:e69977. 10.7554/eLife.69977 PMC8887902 35227374

[47] Barone J , Rossiter HE . Understanding the role of sensorimotor Beta oscillations. Front Syst Neurosci 2021;15:655886. 10.3389/fnsys.2021.655886 PMC8200463 34135739

[48] Kochanski RB , Shils J , Metman LV , Pal G , Sani S . Analysis of movement‐ related beta oscillations in the off‐medication state during subthalamic nucleus deep brain stimulation surgery. J Clin Neurophysiol 2020;36(1):67–73. 10.1097/WNP.0000000000000521 PMC6636622 30418266

[49] Cassidy M , Mazzone P , Oliviero A , Insola A , Tonali P , Di Lazzaro V , Brown P . Movement‐related changes in synchronization in the human basal ganglia. Brain 2002;125(6):1235–1246. 12023312 10.1093/brain/awf135

[50] Joundi RA , Brittain J‐S , Green AL , Aziz TZ , Brown P , Jenkinson N . Persistent suppression of subthalamic beta‐band activity during rhythmic finger tapping in Parkinson's disease. Clin Neurophysiol 2013;124(3):565–573. 23085388 10.1016/j.clinph.2012.07.029

[51] Derrey S , Lefaucheur R , Chastan N , Gérardin E , Hannequin D , Desbordes M , Maltête D . Alleviation of off‐period dystonia in Parkinson disease by a microlesion following subthalamic implantation. J Neurosurg 2010;122(6):1263–1266. 10.3171/2009.10.JNS091032 19877801

[52] Cagnan H , Denison T , McIntyre C , Brown P . Emerging technologies for improved deep brain stimulation. Nat Biotechnol 2019;37(9):1024–1033. 31477926 10.1038/s41587-019-0244-6 PMC6877347

