# Supplementary material for: Cortical and Subthalamic Nucleus Spectral Changes During Limb Movements in Parkinson's Disease Patients with and Without Dystonia
Source: Mov Disord. 2022 Jun 14;37(8):1683–92. doi: 10.1002/mds.29057 (PMC9541849; doi:10.1002/mds.29057)
Supplement: Supplementary file 1 — Figure S1. Correlation between the movement pace and spectral extrema. Above are frequencies of maximum cortical desynchronization during movement compared to rest (Fig. 3). For each cortical region, Pearson correlation is given with p‐values not adjusted for multiple comparisons. Below are frequencies of maximum subcortical synchronization during movement compared to rest. [file MDS-37-1683-s001.pdf]

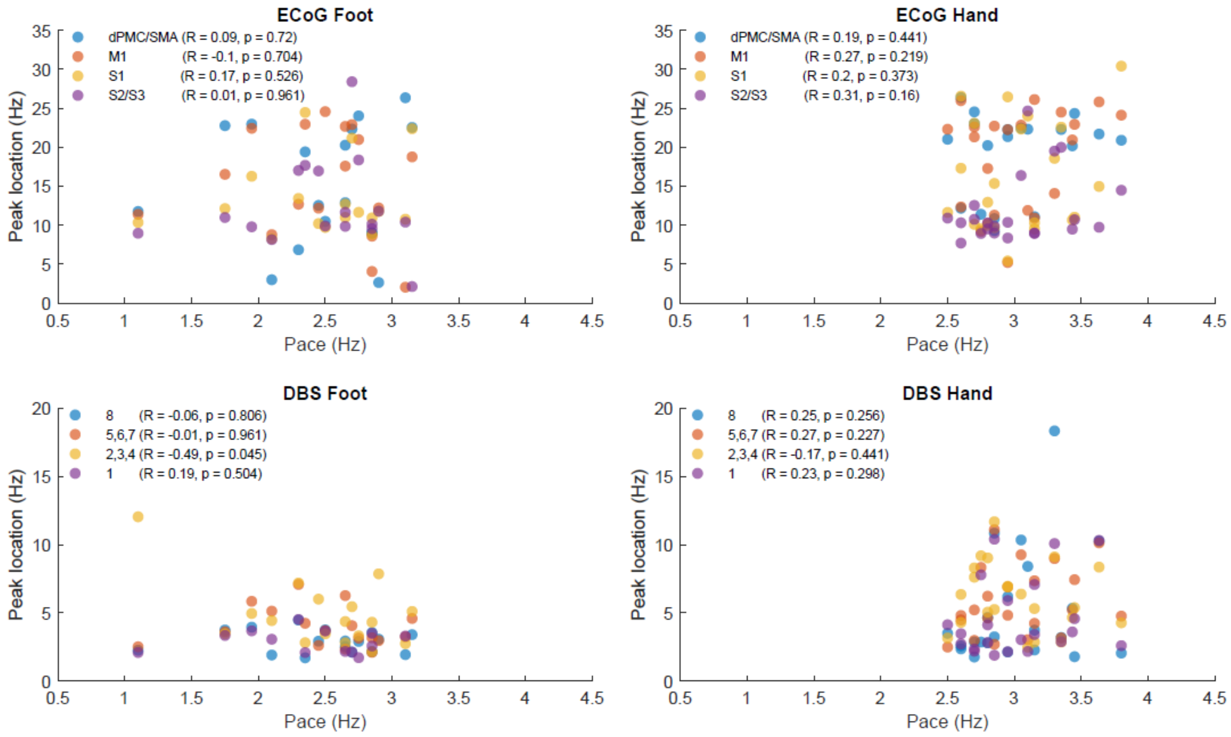

SUPPLEMENTARY FIGURE 1. Correlation between the movement pace and spectral extrema. *Above are frequencies of maximum cortical desynchronization during movement compared to rest (FIGURE 3). For each cortical region, Pearson correlation is given with p-values not adjusted for multiple comparisons. Below are frequencies of maximum subcortical synchronization during movement compared to rest.*
