# Supplementary material for: Cortical and Subthalamic Nucleus Spectral Changes During Limb Movements in Parkinson's Disease Patients with and Without Dystonia
Source: Mov Disord. 2022 Jun 14;37(8):1683–92. doi: 10.1002/mds.29057 (PMC9541849; doi:10.1002/mds.29057)
Supplement: Supplementary file 2 — Figure S2. Averaged cortical and subcortical spectral perturbations during rest and limb movements in PD participants with versus without dystonia determined by different dystonia rating scales. Spectral power from all electrode contacts over ipsilateral sensorimotor cortex (first row) and STN region (second row) are categorized by presence/absence of dystonia historically (UPDRS 4.6), at pre‐operative baseline assessment (BFM), and during DBS surgery (mean ± standard error). Dashed and solid lines represent average spectral power in PD patients with and without dystonia, respectively. Yellow, blue, and red colors correspond to rest, hand, and foot movements. Shaded standard errors are for visual purposes only and do not necessarily reflect the statistical confidence intervals of non‐normal variance. Horizontal colored bars above the plots correspond to frequency intervals with statistically significant differences. During foot movement, we observed statistically significant differences (p < 0.05) between STN low frequency power in PD patients with vs without dystonia when using BFM (4–12 Hz) and surgical exam (4–11 Hz) but not UPDRS. Significant differences (p < 0.05) in STN power were also observed during hand movement when using surgical exam classification (5–15 Hz). No significant differences in STN power were observed during rest for any dystonia classification. Furthermore, there was a significant difference (p < 0.05) in cortical spectral power during hand movement using surgical exam classification (3–25 Hz). Otherwise, there were no observed differences in cortical spectral power between PD patients with and without dystonia. [file MDS-37-1683-s003.pdf]

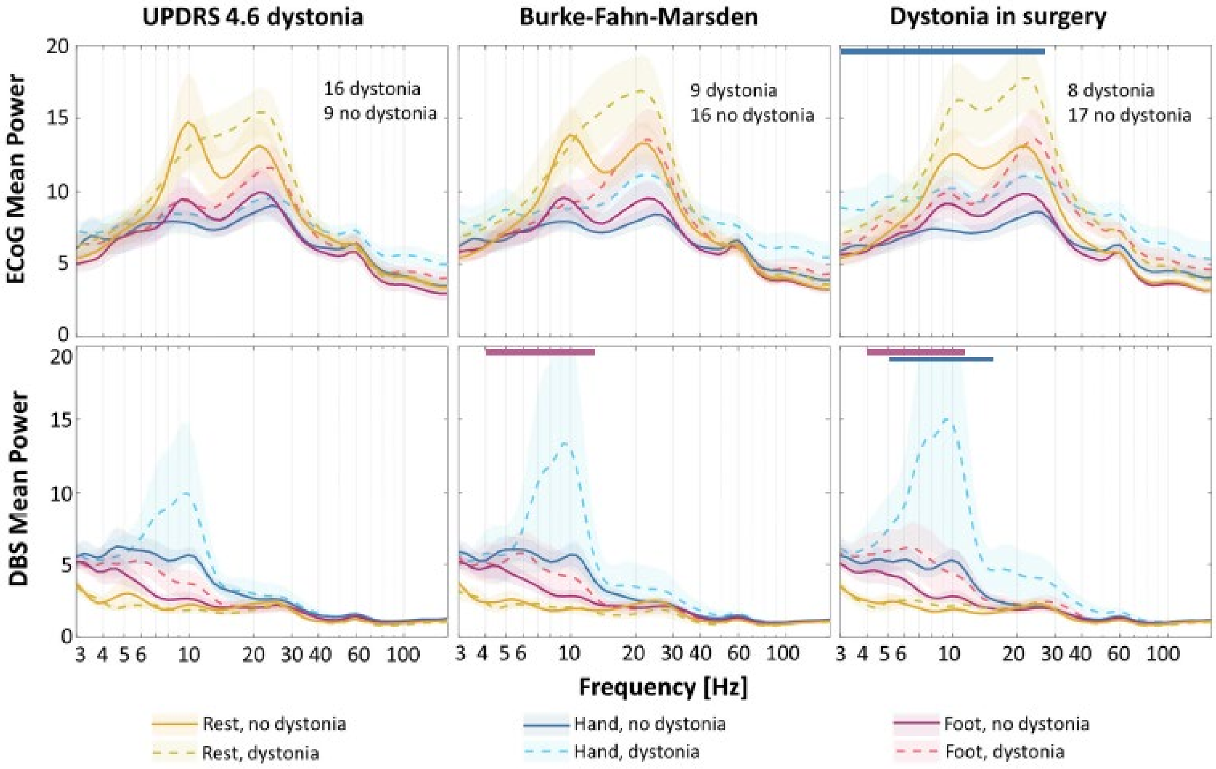

SUPPLEMENTARY FIGURE 2. Averaged cortical and subcortical spectral perturbations during rest and limb movements in PD participants with versus without dystonia determined by different dystonia rating scales. *Spectral power from all electrode contacts over ipsilateral sensorimotor cortex (first row) and STN region (second row) are categorized by presence/absence of dystonia historically (UPDRS 4.6), at pre-operative baseline assessment (BFM), and during DBS surgery (mean ± standard error). Dashed and solid lines represent average spectral power in PD patients with and without dystonia, respectively. Yellow, blue, and red colors correspond to rest, hand, and foot movements. Shaded standard errors are for visual purposes only and do not necessarily reflect the statistical confidence intervals of non-normal variance. Horizontal colored bars above the plots correspond to frequency intervals with statistically significant differences. During foot movement, we observed statistically significant differences ($p<0.05$) between STN low frequency power in PD patients with vs without dystonia when using BFM (4-12 Hz) and surgical exam (4-11 Hz) but not UPDRS. Significant differences ($p<0.05$) in STN power were also observed during hand movement when using surgical exam classification (5-15 Hz). No significant differences in STN power were observed during rest for any dystonia classification. Furthermore, there was a significant difference ($p<0.05$) in cortical spectral power during hand movement using surgical exam classification (3-25 Hz). Otherwise, there were no observed differences in cortical spectral power between PD patients with and without dystonia.*
