# Supplementary material for: Cortical and Subthalamic Nucleus Spectral Changes During Limb Movements in Parkinson's Disease Patients with and Without Dystonia
Source: Mov Disord. 2022 Jun 14;37(8):1683–92. doi: 10.1002/mds.29057 (PMC9541849; doi:10.1002/mds.29057)
Supplement: Supplementary file 3 — Table S1. Patient experiences with dystonia surveyed pre‐surgery. [file MDS-37-1683-s002.pdf]

General description of dystonia	Activities that worsen dystonia	Body part affected by dystonia
'All the time when meds wear off.'	'No specific activities.'	'Both feet. Toes curl.'
'Unclear to participant. It seems random.'	'Standing, walking, resting.'	'Left, middle, fingers, and toes on right foot. Right fingers will draw.'
'Leg morning and evening (morning before medicines work).'	'Ocular is brought on by bright lights. Leg activity is brought on if he thinks about it'	'Leg and eye'
'Every 4 hours when meds wear off'	'She experiences it at rest.'	'Right toes. Right leg. Both wrists. Her neck.'
'Shortly after dose of medicine until it starts wearing off.'	'Sitting, at rest'	'Left foot'
'Only notice it when sitting on couch watching TV at the end of the day'	'Only notice it when resting'	'Left and right feet areas affected'
'When tired'	'Walking will bring it on'	'Left foot'
'Does not happen at specific time of day'	'Sitting and rest'	'Both legs'
'1st thing in the morning when meds are off.'	'At rest, also hands will activate if reading or holding something; Foot at rest, with meds off.'	'Both feet, both hands/wrists and neck.'
'Afternoon'	'At rest'	'Neck'
'When medicines are off'	'Anything that requires continuous effort/force'	'Right side hand and toes'
'When medicines wear off'	'Rest'	'Neck, trunk, right arm and right foot'
'All the time.'	'Always.'	'Left toes and neck.'
'Starts after participant takes the first dosage of medicine'	'At rest'	'Left leg'
'Night, afternoon'	'Lack of sleep, lack of exercise.'	'Right shoulder, hip, foot'
'Occurs often'	'Walking, anxiety'	'Right foot'
'Does not occur often'	'Rest'	'Left arm'

SUPPLEMENTARY TABLE 1. Patient experiences with dystonia surveyed pre-surgery.
